# The Use of Artificial Intelligence (AI) in the Radiology Field: What Is the State of Doctor–Patient Communication in Cancer Diagnosis?

**DOI:** 10.3390/cancers15020470

**Published:** 2023-01-12

**Authors:** Alexandra Derevianko, Silvia Francesca Maria Pizzoli, Filippo Pesapane, Anna Rotili, Dario Monzani, Roberto Grasso, Enrico Cassano, Gabriella Pravettoni

**Affiliations:** 1 Applied Research Division for Cognitive and Psychological Science, IEO European Institute of Oncology IRCCS, 20141 Milan, Italy; 2 Department of Oncology and Hemato-Oncology, University of Milan, 20122 Milan, Italy; 3 Breast Imaging Division, IEO European Institute of Oncology IRCCS, 20139 Milan, Italy; 4 Department of Psychology, Educational Science and Human Movement, University of Palermo, 90128 Palermo, Italy

**Keywords:** artificial intelligence, communication, decision-making, patient empowerment

## Abstract

**Simple Summary:**

Artificial Intelligence (AI) has been increasingly used in radiology to improve diagnostic procedures over the past decades. The application of AI at the time of cancer diagnosis also creates challenges in the way doctors should communicate the use of AI to patients. The present systematic review deals with the patient’s psycho-cognitive perspective on AI and the interpersonal skills between patients and physicians when AI is implemented in cancer diagnosis communication. Evidence from the retrieved studies pointed out that the use of AI in radiology is negatively associated with patient trust in AI and patient-centered communication in cancer disease.

**Abstract:**

Background: In the past decade, interest in applying Artificial Intelligence (AI) in radiology to improve diagnostic procedures increased. AI has potential benefits spanning all steps of the imaging chain, from the prescription of diagnostic tests to the communication of test reports. The use of AI in the field of radiology also poses challenges in doctor–patient communication at the time of the diagnosis. This systematic review focuses on the patient role and the interpersonal skills between patients and physicians when AI is implemented in cancer diagnosis communication. Methods: A systematic search was conducted on PubMed, Embase, Medline, Scopus, and PsycNet from 1990 to 2021. The search terms were: (“artificial intelligence” or “intelligence machine”) and “communication” “radiology” and “oncology diagnosis”. The PRISMA guidelines were followed. Results: 517 records were identified, and 5 papers met the inclusion criteria and were analyzed. Most of the articles emphasized the success of the technological support of AI in radiology at the expense of patient trust in AI and patient-centered communication in cancer disease. Practical implications and future guidelines were discussed according to the results. Conclusions: AI has proven to be beneficial in helping clinicians with diagnosis. Future research may improve patients’ trust through adequate information about the advantageous use of AI and an increase in medical compliance with adequate training on doctor–patient diagnosis communication.

## 1. Introduction

In the last four decades, medical technology has seen a shift in the development of Artificial Intelligence (AI) which is commonly defined as “a field of computer science that develops systems able to perform tasks commonly associated with intelligent human beings” [1]. AI refers to machines or systems that can act for themselves and make decisions when faced with new situations such as problem-solving or decision-making systems. AI applications include machine learning (ML), natural language processing, automated speech recognition, deep learning (DL), computer vision, and radiomic [2,3]. Particularly, ML, introduced by Arthur Samuel in 1959, defines a field of artificial intelligence where a computer learns automatically from data accumulation, whereas DL emerged as a promising approach for image processing [4], allowing the system to recognize patterns and make predictions [5]. The use of AI demonstrated significant progress in image-recognition tasks [6]. Indeed, AI is one of the fastest-growing areas of informatics and computing with great relevance to healthcare and radiology. Some media headlines claiming doctors’ better performances have fueled hype among the public and the press for accelerated implementation of AI techniques. Examples include: “Google says its AI can spot lung cancer a year before doctors” and “AI is better at diagnosing skin cancer than your doctor, study finds” [7,8].

Considering the radiology community, there is a relevant interest in applying AI to improve workflow applications and patient care. AI is considered an optimizing tool to assist the radiologist in detecting suspicious findings in imaging exams, making the diagnosis, choosing a personalized patient protocol, tracking the patient’s dose parameters, providing an estimate of the radiation risks [9,10], and also minimizing diagnostic errors. Indeed, despite human intuition on visual perception providing a faithful representation of the world, we often miss salient events in our environment when we are focused on something else. This phenomenon is known as inattentional blindness, i.e., the failure to notice an unexpected but fully visible stimulus when attention is engaged in another task [11]. While enhanced global processing ability generally allows expert radiologists to rapidly detect abnormalities, including unexpected ones [12], inattentional blindness may provide insight into ways to address a growing concern in radiology: missed but clinically significant incidental findings, which are abnormalities in medical images that are unrelated to the patient’s main symptomatology and that may even be detected in asymptomatic patients [13]. Furthermore, AI in the medical field might also result in significant support for radiologists’ cognitive fatigue, which is often a consequence of their daily demanding medical practice. Medical doctors support the use of AI algorithms as aiding tools for precision medicine. Sarwar and colleagues [14] reported that 75% of 487 interviewed physicians from 54 countries showed positive attitudes toward AI and expressed interest in AI as a diagnostic tool to improve workflow efficiency and quality assurance. A 2018 study pitted dermatologists against a computer that had been trained to differentiate between cancerous skin lesions and benign ones [15]. The results showed dermatologists were only 86.6% accurate at diagnosing skin cancer, while the computer was able to diagnose issues with a 95% accuracy. Another study [16] on AI diagnostic accuracy using endoscopic images for the detection of cancer or neoplastic lesions and the classification of lesions (neoplastic vs. nonneoplastic) in the gastrointestinal tract determined that AI was accurate but had a lower performance compared to the highly accurate endoscopist.

For all these premises, AI holds great promise for the oncology field, and it can be especially useful as a means for mammography screening [17,18]. However, although AI can provide detailed quantifications of tissues on imaging examinations, which can be used for diagnostic, prognostic, and treatment purposes [19], this technology should not be currently used as a standalone medical device, but it should be considered the combination of software and radiologists [20,21,22]. Furthermore, AI should never outweigh the development of rigorous evidence-based medical practice [15].

Considering the implementation of AI in radiology clinical practice, multiple steps from routine screening based on risk factors to communication reports should be targeted. On one hand, radiologists must play a leading role in developing and validating AI applications for medical imaging; on the other hand, they also must manage the risk that the medical–patient interaction might become more impersonal [23]. To prevent this, patients’ points of view should be taken into consideration. The European Union has indeed recognized the problem that algorithm-based medical decision-making poses in this regard and has published a landmark paper highlighting the need for explanations of computerized decision-making in the medical field so that patients can effectively understand the crucial role AI can play in their health [24,25]. The solution is found in the concept of explainable AI (XAI), which is attracting increasing interest in the scientific community [26]. Communication can be seen as a pivotal ingredient in medical care, and XAI might provide a patient-friendly explanation of biomedical decisions based on ML. Particularly, XAI would be highly valuable in the oncology field, where it is essential to consider not only the purely medical aspects but also the patient’s psychological and emotional dimensions [27]. Technological aspects of AI systems are largely described by the current literature in different health sectors. However, the patient’s standpoint of AI to make decisions on their health is often neglected. Scarce communication between patients and clinicians about the potential benefits of AI is likely to cause to patients’ mistrust of such a promising tool. Indeed, most patients perceive an AI-aided diagnosis as not completely reliable [28,29]. One of the reasons behind this mistrust can be identified in the “Third Wheel Effect” [30], whereby the patient considers the AI as an unnecessary intrusion rather than an added value. Specifically, patients may have a perception that their relationship with their doctor will suffer because of the “third wheel”, which might then result in “decision paralysis”, risk of decision-making delays, “Confusions of the Tongues” and ambiguity.

Overall, current evidence regarding patients’ perceptions of AI in radiology and related communication issues is very limited. Since this field is under-explored, this review aims to discuss the use of AI in radiology and the challenges that AI poses in doctor–patient communication. Therefore, the authors propose future research directions to implement doctor–patient communication skills and to support patients’ understanding of AI at the time of their cancer diagnosis.

## 2. Materials and Methods

### 2.1. Search Strategy

**Figure 1:**
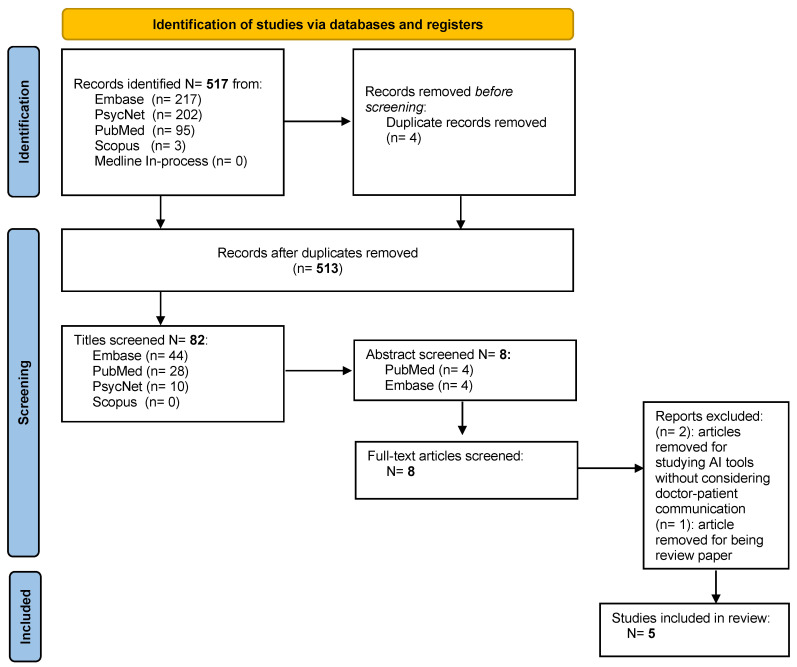
Preferred Reporting Item for Systematic Review (PRISMA) study selection flow diagram.

A systematic review of the literature was performed to identify the use of AI in the field of radiology in doctor–patient communication when communicating the diagnosis of cancer. The systematic review was conducted and reported following the Preferred Reporting Items for Systematic Reviews and Meta-Analyses (PRISMA) guidelines [31] (Figure 1). The protocol for this systematic review has not been registered. Digital literature databases, including PubMed, EMBASE, Medline, Scopus, Psycnet, and Medline In-process were searched from 1990 to 2021. Only studies published during the last decade were considered since they are more likely to report current developments in IA in the radiological field and psychological aspects such as the importance of doctor–patient communication. MeSH was used to identify label terms to extract as many articles as possible related to the topic. The keywords and descriptors used in any field were “artificial intelligence” OR “intelligence machine” AND “communication” AND “radiology” AND “oncology diagnosis”.

### 2.2. Inclusion and Exclusion Criteria

All publication types and all study designs were included, with no language or age restriction. The following inclusion criteria were applied: studies that reported the development of AI in radiology in cancer diagnosis; studies with patients’ perception of artificial intelligence; studies highlighting the oncological diagnosis communication; studies with patients’ point of view on the oncological doctor–patient communication of the AI diagnosis; the use of AI in screening mammography. Medical AI studies without considering doctor–patient communication and papers dealing with the use of AI in other fields were excluded.

### 2.3. Screening and Data Extraction

Two independent reviewers undertook all titles and abstract screening (A.D. and S. F. M. P.) resulting from the literature search for inclusion and exclusion criteria. Disagreements were solved by a discussion with all the members of the research team.

## 3. Results

In total, 517 publications were identified, and of those, 4 duplicates were removed before the initial screening. Then, 431 articles based on the screening of titles and 74 articles based on the screening of abstracts were excluded. Eight full-text articles were assessed for eligibility. Three articles were excluded, two were removed for studying AI tools without considering doctor–patient communication, and one article removed for being a review paper. Following the full-text screening, five studies met the inclusion criteria.

### 3.1. Features of the Studies

The study findings are summarized in Table 1. The overall sample size of the studies includes 939 participants. The majority of the participants were over 18 years old and female. Among the retrieved studies, one adopted a longitudinal design, one used a semi-structured interview, and three were qualitative studies regarding the patient’s attitude toward AI. Overall, the included studies reported limited data on the characteristics of the patients (diagnosis, cancer stage, etc.). Details of the retrieved studies are reported in Appendix A.

### 3.2. Synthesis of the Results

The most relevant and recurrent variables across studies concerning patients’ attitudes toward AI and issues in doctor–patient communication are summarized in Table 2.

Ongena et al. [32] conducted a longitudinal study using an Internet survey for the social science panel on the Dutch population to investigate the general population’s view on the use of AI for the diagnostic interpretation of screening mammograms. The study included 922 women from 16 to 75 years old. Five items were measured to investigate the patient’s attitude toward AI in mammography: “Necessity of a human check”, “AI as a selector for second reading”, “AI as a second reader”, “Developer is responsible for error”, “Radiologist is responsible for error”. No standardized questionnaires were used, but a 5-point Likert scale was developed ad hoc to collect patients’ agreement or disagreement. The authors analyzed the different items with the variable “education”, finding that there were different patients’ perceptions between those who have a high level of education and those who do not. Results highlighted that those who find a human check of mammograms necessary tend to prefer a personal interaction in discussion results and consider AI less efficient because of lower education. On the contrary, those who find a human check as neutral tend to view personal interaction in discussing results as less important and consider AI more efficient, keeping a positive attitude towards health technology. Adams et al. [33] hosted a patient engagement workshop and employed qualitative analysis to determine the initial patient perceptions, patient priorities for AI use cases, and patient-identified evaluation metrics. Qualitative interviews were conducted with 17 patients (11 female and 6 male, age and diagnosis were not indicated). The authors identified common themes or patterns from text data. The initial perceptions of AI captured four themes: (1) “Fear of the unknown”, (2) “Trust”, (3) “Human connection” and (4) “Cultural acceptability”. Patients’ perceptions of AI were shaped by popular media and science fiction. Some participants expressed fear or described AI as an unknown scary instrument. Trust or lack of trust was the consequence of fear of the unknown AI tool in radiology. For most of the participants, a lack of knowledge also represented a lack of trust in AI, while others displayed a willingness to trust outputs from AI, which might achieve the most accurate information. Furthermore, some participants were concerned about the lack of human connection and that AI might enhance the necessity for “human empathy” and the human “ability to understand with flexibility”. Overall, the main result was that all participants underlined the importance of an understandable way to explain the AI results because in some cases medical language emerged as either too difficult or unclear. Indeed, participants emphasized the need to fully understand their imaging results to be engaged in their care and have more productive conversations with their physicians. Carter et al. [34] compiled a narrative review concerning the ethical point of view of doctor–patient communication in radiology using AI. Indeed, patients understand little about health technologies and perhaps do not understand AI systems. Mendelson [35] facets the potential and limitations of AI in breast imaging. The author stressed the importance of the potential of AI in radiology concerning the improvement of the workflow of the algorithms of AI and the outcome analyses that are advancing in the last decades. The main role of the high-tech in AI was the use of imaging data in high quality and quantity, so that AI can support breast imagers in diagnosis and patient management. The importance of physicians’ knowledge and expertise was specifically stressed in survival phase decision-making.

Kapoor et al. [36] provided an overview of available tools and developed considerations on the workflow applications of AI. In this work, AI is proposed to optimize patient scheduling, improve worklist management, and help radiologists interpret diagnostic studies. AI applications were described as multiple and complex processes ranging from routine screening to report communication, with several implementation steps. Kapoor et al. [36] highlighted the relevance of the final step in the diagnostic imaging chain that concerns the report communication. The authors described this process as an underrecognized area in which quality of care issues can arise. Moreover, ML algorithms can identify specific disease entities in radiology reports, and can be used to accurately identify tailored follow-up recommendations. The authors concluded that data in feedback reports could be used to ensure appropriate closed-loop communication to monitor radiologist variation in follow-up recommendations [37].

## 4. Discussion

Our review explored the implications of using AI on doctor–patient communication at the time of cancer diagnosis in the field of radiology. According to our findings, this is still a low-investigated topic in the literature.

The use of AI in healthcare involves not only technical issues but also ethical, psycho-cognitive, and social-demographic considerations of presenting patients with cancer with the presence of AI at the time of the diagnosis. Trust, Accountability, Personal interaction, Efficiency, and General attitude toward AI were identified as five core areas by Ongena et al. [38]. The variables that merge such aspects of patients’ attitudes to using and communicating diagnosis with AI are education and knowledge. Accordingly, the authors showed that participants who have lower education are less supportive of AI, and those who have thought AI to be less efficient have a more negative attitude toward AI. Therefore, it is possible to consider that those who do not have a good understanding of the way AI works tend to have a negative attitude toward its effectiveness and less trust in its potential. Moreover, those who mistrust the diagnostic accuracy of AI as well as are not well educated tend to seek interpersonal interaction with doctors much more than those who were neutral about the efficacy of AI. One of the items, the “Necessary of a human check”, relates very closely to the importance of doctor–patient communication, focusing on the need to integrate two aspects: the use of high-tech in diagnosis and the need for human–doctor communication about the exam results. This point underlines the pivotal role of the doctor’s communication in a circumstance of little knowledge about a new tool in healthcare such as AI. Starting from the premise that the current evidence regarding patients’ experiences, perceptions, and priorities for artificial in radiology are limited, Adams et al. [33] investigated a patient’s knowledge and perceptions on the use of AI in a care setting. Despite the methodological difference from the previous article, some fundamental and very similar themes emerged. In this case, there are four thematic cores: fear of the unknown, trust, human connection, and improving communication. Therefore, on the patients’ side, these aspects are a strong issue of where to place trust. These difficulties in participants’ understanding of the use of new technologies, such as AI in radiological diagnosis, imply the need for more human connection, and at least the necessity to improve the quality of communication with the doctors. Indeed, some participants were concerned about the lack of human connection and that AI may emphasize the necessity of “human empathy”. The qualitative interviews showed that patients felt the topic of “improving communication” was a priority for AI use cases. This result may reflect again the importance of doctor–patient communication throughout the healthcare process from examination scheduling to diagnosis communication. In addition to the complexity of the different layers of AI involved such as DL and ML, there is a strong debate about the medical decision-making process with such a tool. Carter et al. [34] highlighted that patients are still very hesitant when faced with AI outputs, as the image of the “machine” conveys the idea of something that can make mistakes. On the physician’s side, AI has implications for human capacities. Firstly, AI could lead to a change in clinicians’ skills. Indeed, they are more likely to lose capabilities they do not regularly use, for example, if they read fewer mammograms. A second point about professional responsibility concerns automation bias which means that humans tend to accept machine decisions, even when they are wrong [39]. To overcome these risks, it is necessary to train clinicians to avoid or lower automation bias. Mendelson [35] focused not only on patients’ perceptions and their knowledge about AI, but also on the need for physicians to empower their skills in communication. Although doctors may know very well their medical and scientific language and the functions of their technological tools, they do not systematically train their skills in diagnosis communication, especially when they use AI tools. Recently, a systematic literature review addressed an important gap in cancer care focusing on the impact of Health Information Technology (HIT) on doctor–patient communication. Studies showed that some types of HIT can increase patients’ confidence and support their active involvement in the care processes while maintaining a good relationship with the healthcare team (38). Therefore, a patient’s knowledge of diagnostic tools is as important as a physician’s communication skills. Kapoor et al. [36] shed light on the concept of closed-loop communication. The authors described that sometimes there is variability in radiologists’ language and follow-up recommendations and that machines using AI collocated in different hospitals can have different outcomes. The divergence of outcomes requires doctors to understand what is wrong with the machines and discuss the meaning of the discrepant results, while their communication remains in a closed loop, not engaging patients. It is well known that the effectiveness of medical treatment depends on the quality of the patient–clinical relationship [40] and the use of AI in the field of radiology poses challenges in doctor–patient communication at the time of the diagnosis. Therefore, implementing the doctor–patient communication of AI results and issues may change the patient’s choice in their health.

### 4.1. Limitations

Overall, the literature on the topic is scarce. Furthermore, there is high heterogeneity in the methodologies of studies, which range from a longitudinal study to a narrative review, including qualitative analysis. The heterogeneity of studies posed challenges to the systematization of the results. It also shed light on the fact that the main topic, assessed over time and despite different methods, produced similar results. Finally, the heterogeneity of the samples rendered it difficult to define AI attitudes in specific subsamples of patients or specific moments of the cancer care pattern.

### 4.2. Future Directions

Future research may consider some useful steps in applying AI bearing in mind patients’ psycho-cognitive perspectives. We propose the acronym AIR-IUT to highlight the three main steps to be considered in the application of AI in the field of radiology and future studies dealing with the patient’s experience of the application of AI. The acronym stands for the fact that in the field of Artificial Intelligence in Radiology, the process is to Inform patients to Understand and Trust the use of AI. Future interventions should consider implementing the use of digital platforms with illustrative videos to inform patients, offering reliable educative means that might be delivered in the waiting rooms. Indeed, involving patients with digital interaction could increase compliance, reduce the fear of the unknown about health technology and psychological feelings, and improve patients’ decision-making at the time of treatment, since they are actively involved and informed at the screening time [41]. Concurrently, a training course to enhance doctor–patient communication skills at the time of diagnosis may be developed. Such a course should help clinicians to adopt patient-friendly language (i.e., jargon words must be explained or replaced by simpler words) and an empathetic approach, entailing particular attention to the patient’s psychological well-being.

## 5. Conclusions

In conclusion, doctors should sharpen their communication skills when AI is involved in diagnosis, and patients should be engaged in the process mainly by being informed on the functioning of medical tools used to formulate their diagnosis. One of the most evident elements from the retrieved studies is that patients do not know what AI is and this lack of knowledge affects trust and doctor–patient communication. Since patients should be empowered and tailor informed at all phases of their clinical journey, they should ideally know which diagnostic tools are used by their clinicians and the way they work. Given the outstanding AI’s potential, we believe that informing patients about its progress in our field will help them to be more trusting towards it.

## Figures and Tables

**Table 1:** Summary of the study sample patients’ characteristics, attitudes toward Artificial Intelligence.

References	Patient Characteristics			Attitude toward AI		Patient’s Knowledge and Point of View on AI
	Populations	N	Average Age (SD)	Investigated	Language Population	
Ongena et al., 2020 [32]	Breast cancer screening	922	±45	Trust Accountability Personal interaction Efficiency The general attitude toward AI	German	Those who have lower education are less supportive of AI Those who think AI is less efficient had a more negative attitude toward AI
Adams et al., 2020 [33]	/	17	/	Fear of the unknown Trust Human connection Improving communication	English	AI was shaped and viewed as “science fiction”
Carter et al., 2019 [34]	Breast cancer	/	/	Ethical Legal Social implications	English	No deep understanding of the way health technologies work
Mendelson, 2019 [35]	Breast cancer	/	/	Potentials Limitations	English	Education in AI for patients Empowerment skills in doctor–patient communication
Kapoor et al., 2020 [36]	/	/	/	Workflow applications of AI in radiology	English	Closed-loop communication of critical radiology results

**Table 2:** Main findings on patients’ psycho-cognitive attitudes toward Artificial Intelligence and communication issues.

References	Methods	Analysis	Main Variables
Ongena et al., 2020 [32]	Internet Survey with ad hoc 5-point Likert Scale	Quantitative analysis	Patients’ education levels shape trust and attitudes toward AI (low education is associated with low trust)
Adams et al., 2020 [33]	Patient engagement Workshop and interviews	Qualitative analysis (thematic analysis)	Trust is linked to the fear of the unknown uses of AI in radiology and the lack of human connections and empathy
Carter et al., 2019 [34]	Narrative review and perspective	Analysis of the ethical issues in doctor–patient communication	Knowledge and understanding of the way AI works are pivotal for the ethical use of AI
Mendelson, 2019 [35]	Narrative review and perspective	Analysis of the pros and cons of using AI in breast cancer imaging	Knowledge and education about AI for patients are as important as the empowerment of skills in communication for physicians
Kapoor et al., 2020 [36]	Overview of the applications of AI in radiology	Qualitative synthesis	Closed-loop communication to provide improved and personalized feedback for patients

## Appendix A

**Table A1:** Summary of General Characteristics of Included Studies.

Title	URL	Resource	Type	Identifiers	Db
(1) Workflow Applications of Artificial Intelligence in Radiology and an Overview of Available Tools	doi.org/10.1016/j.jacr.2020.08.016	PubMed	Narrative review	PMID: 33153540	MeSH-PubMed
(2) Artificial Intelligence in Breast Imaging: Potentials and Limitations	doi.org/10.2214/AJR.18.20532	PubMed	Narrative review	PMID: 30422715	MeSH-PubMed
(3) Patient Perspectives and Priorities Regarding Artificial Intelligence in Radiology: Opportunities for Patient-Centered Radiology	doi.org/10.1016/j.jacr.2020.01.007	PubMed	Qualitative	PMID: 32068006	MeSH-PubMed
(4) The ethical, legal and social implications of using artificial intelligence systems in breast cancer care	doi.org/10.1016/j.breast.2019.10.001	PubMed	Narrative review	PMID: 31677530	MeSH-PubMed
(5) Artificial intelligence in screening mammography: A population Survey of Women’s Preferences	doi.org/10.1016/j.jacr.2020.09.042	PubMed	Longitudinal study	PMID: 33058789	MeSH-PubMed
